# Context-Specific and Immune Cell-Dependent Antitumor Activities of α1-Antitrypsin

**DOI:** 10.3389/fimmu.2016.00559

**Published:** 2016-12-07

**Authors:** Ofer Guttman, Gabriella S. Freixo-Lima, Ziv Kaner, Yotam Lior, Peleg Rider, Eli C. Lewis

**Affiliations:** ^1^Department of Clinical Biochemistry and Pharmacology, Faculty of Health Sciences, Ben-Gurion University of the Negev, Beer-Sheva, Israel

**Keywords:** CD8^+^ T lymphocytes, nitrosylation, reactive oxygen species, tumor-associated macrophages, tumor immunology

## Abstract

α1-antitrypsin (AAT), a circulating glycoprotein that rises during acute phase responses and healthy pregnancies, exhibits immunomodulatory properties in several T-cell-dependent immune pathologies. However, AAT does not directly interfere with T-cell responses; instead, it facilitates polarization of macrophages and dendritic cells towards M2-like and tolerogenic cells, respectively. AAT also allows NK cell responses against tumor cells, while attenuating DC-dependent induction of autoimmune NK cell activities. Since AAT-treated macrophages bear resemblance to cancer-promoting tumor-associated macrophages (TAMs), it became imperative to examine the possible induction of tumor permissive conditions by AAT. Here, AAT treatment is examined for its effect on tumor development, metastatic spread, and tumor immunology. Systemic AAT treatment of mice inoculated with B16-F10 melanoma cells resulted in significant inhibition of tumor growth and metastatic spread. Using NK cell-resistant RMA cells, we show that AAT interferes with tumor development in a CD8+ T-cell-dependent manner. Unexpectedly, upon analysis of tumor cellular composition, we identified functional tumor-infiltrating CD8+ T-cells alongside M1-like TAMs in AAT-treated mice. Based on the ability of AAT to undergo chemical modifications, we emulated conditions of elevated reactive nitrogen and oxygen species. Indeed, macrophages were stimulated by treatment with nitrosylated AAT, and IFNγ transcripts were significantly elevated in tumors extracted soon after ischemia-reperfusion challenge. These context-specific changes may explain the differential effects of AAT on immune responses towards tumor cells versus benign antigenic targets. These data suggest that systemically elevated levels of AAT may accommodate its physiological function in inflammatory resolution, without compromising tumor-targeting immune responses.

## Introduction

α1-Antitrypsin (AAT) is currently gaining recognition as an immunomodulatory agent capable of improving outcomes in a variety of immune conditions (1, 2). AAT levels rise physiologically in the circulation during acute phase responses, as well as during the third trimester of pregnancy. AAT is routinely infused to individuals who lack sufficient circulating AAT due to a genetic deficiency (AATD) (3). According to preclinical studies, treatment with AAT induces long-term tolerance to allogeneic islet transplants (4, 5) and alleviates aspects of autoimmune diabetes (6), graft-versus-host disease (GvHD) (7), inflammatory bowel disease (8), and multiple sclerosis (9). Some of these target diseases are presently assessed in multiple Phase II clinical trials. While AAT has been thoroughly documented as an inhibitor of inflammatory serine proteases, primarily neutrophil elastase, cathepsin G, and proteinase 3 (1, 2), evidence suggests a mechanism of operation independent of protease inhibition that extends toward immune-oriented innate cellular targets (1, 2). As such, evidence points to effects dependent upon several environment-dependent covalent modifications of AAT, such as that take place in environments rich in reactive oxygen species and/or reactive nitrous species; indeed, protease targets of naïve AAT (1, 5), oxidized AAT (5, 10, 11), and nitrosylated AAT (S-NO-AAT) (12) appear to be largely separate.

Dendritic cells (DCs) and macrophages are among the most incessant cellular targets of AAT (13, 14); treating these cells with AAT, concomitant with inflammatory stimulation, results in reduced inflammatory activation profiles and an apparent redirection of the mode of activation toward an immunomodulatory phenotype (13, 14). Specifically, AAT-treated animals that underwent allogeneic skin and islet transplantation exhibited DCs with a semi-mature DC (smDC) phenotype (14); these DCs secreted anti-inflammatory cytokines, such as IL-1 receptor antagonist (IL-1Ra) (15), IL-10 (14), and transforming growth factor β (TGFβ) (4), and promoted the differentiation of regulatory T cells (Tregs) (14). Tumor-associated DCs (TADCs) are DCs present in the periphery of solid tumors, which have been suggested to act as immune suppressors (16). While these cells seem to bear certain similarities to AAT-treated DCs, the influence of AAT over TADCs has yet to be examined. AAT-treated macrophages exhibit a similar trend by polarizing toward the M2-like profile in the presence of AAT and various inflammatory stimuli (13). Importantly, while AAT treatment does not directly affect isolated T (4, 5) or natural killer (NK) (17) cell responses, AAT *indirectly* promotes Treg differentiation (14) and alters NK cell responses in mixed cultures, thus diminishing cytotoxic CD8^+^ effector T cell expansion (14). AAT further acts indirectly through DC-mediated mechanisms to divert NK cells away from non-authentic threats (17). It was recently established that while isolated NK cell responses against tumor cells remain intact in the presence of AAT, NK cell activities are *indirectly* modulated by reduced cross-activation signals from stimulated DCs (17).

While literature regarding the precise influence of AAT over tumor development is strikingly scarce, several studies have established that AAT treatment inhibits both tumor development and tumor angiogenesis in mice (18), and that AAT treatment of MCF-7 breast cancer cells had resulted in reduced tumor cell proliferation rates and invasiveness (19–21). Nonetheless, these data do not address the possible influence of AAT over leukocyte–tumor interactions that are of critical importance in tumor immunology.

Versatility and plasticity are among the hallmarks of macrophages (22–24). When exposed to various cytokines and environmental factors, macrophages may polarize and acquire either pro- or anti-inflammatory characteristics. Pro- and anti-inflammatory macrophages are often termed M1-like and M2-like macrophages, respectively (22–24). M1-like and M2-like macrophages represent the extremes of a wide continuum of possible polarization states, with numerous intermediary states, and suitable stimulation may redirect polarized macrophages across the spectrum. Indeed, macrophage repolarization has been demonstrated to be involved in ameliorating disease progression in models of type 1 diabetes (25), inflammatory bowel disease (26–28), and multiple sclerosis (29).

M2-like macrophages share numerous characteristics with tumor-associated macrophages (TAMs) that hold a critical role in tumor progression (30–33). TAMs are derived from blood-borne inflammatory monocytes that infiltrate into the tumor tissue and polarize toward the M2-like phenotype (34, 35). AMs engage in an intricate bidirectional cross-talk with local tumor cells (31, 36–38), leukocytes (39–42), fibroblasts (43), and endothelial cells (44). TAMs facilitate tumor progression by promotion of angiogenesis (44–46), secretion of growth factors such as TGFβ and vascular endothelial growth factor (VEGF) (30–33), and suppression of antitumor lymphocytes (31, 47–49). In particular, CD8^+^ T cells are locally inhibited by TAM-derived IL-10 and TGFβ and are compromised by TAM-induced Tregs (31, 50, 51). In contrast, M1-like macrophages elicit cytotoxic responses from CD8^+^ T cells, through the secretion of IL-12, IL-18, type I IFNs, and tumor necrosis factor α (TNFα) (31, 52, 53), and can directly kill tumor cells through the release of nitric oxide (NO) (53). Importantly, treatment of pro-tumor TAMs with IFNγ (54) or with augmentation of the NF-κB pathway (55) has been shown to reverse TAM polarization and induce a pro-inflammatory phenotype.

In the present study we employ several *in vivo* tumor inoculation models and assess their outcomes under AAT therapy. We specifically examine intra-tumor leukocyte composition and activation profiles and the contribution of AAT-modified innate cells toward effective CD8^+^ antitumor T cell responses. While assessment of unmodified human α1-antitrypsin (hAAT) has uniformly suggested anti-inflammatory characteristics, factors prevalent in tumor environments such as hypoxic conditions (11) or elevated concentrations of NO (12) have been suggested to alter the effects of AAT. Therefore, covalent environment-based modifications of AAT are addressed using settings that incorporate nitrosylated AAT, as well as an ischemia–reperfusion (IRP) model that examines the function of AAT in a hypoxic environment characteristic of tumors. These models serve to investigate the hypothesis that AAT may attain an appropriate context-specific and immunocyte-specific activity profile so as to enhance immune antitumor responses.

## Materials and Methods

### Mice

Wild-type (WT) C57BL/6 mice were purchased from Harlan Laboratories (Rehovot, Israel). Transgenic hAAT^+/+^ mice with C57BL/6 background were engineered as described (56) and bred in-house. All animal studies were approved by the Animal Care and Use Committee, Ben-Gurion University of the Negev. Female 6- to 10-week-old mice were used in all experiments.

### Cell Line Cultures

The B16-F10 cell line (murine melanoma cell line, no. CRL-6475) was purchased from American Type Cell Cultures (ATCC, Manassas, VA, USA), while the RMA and RMA-S cell lines (murine lymphoma origin) were generously provided by A. Porgador (Ben-Gurion University of the Negev). RMA-S cells were originally generated by permanent silencing of MHC class I expression in RMA cells; they are therefore non-sensitive to MHC class I-restricted CD8^+^ T cells, but sensitive to NK cells due lack to of MHC class I-dependent inhibition of NK cells (57, 58). Cells used in all experiments were from the first six passages. B16-F10 cells were cultured in DMEM medium, and all other cell lines were cultured in RPMI-1640 medium, supplemented with 10% heat-inactivated FBS, 2 mM l-Glutamine, 50 U/ml penicillin, and 50 μg/ml streptomycin (all from Biological Industries, Beit HaEmek, Israel) under standard conditions. Where stated, cell cultures were treated with indicated concentrations of human serum-purified hAAT (Glassia™, Kamada Ltd., Nes-Ziona, Israel). Cells were routinely verified to be mycoplasma-free.

### Generation and Activation of and BMDMs

Bone marrow-derived macrophages (BMDMs) were generated by flushing of murine tibia and femur bones with sterile PBS and culturing of bone marrow cells in complete RPMI-1640 medium supplemented with 50 μM β-mercaptoethanol (Sigma-Aldrich, Rehovot, Israel). Cells were cultured with granulocyte–macrophage colony-stimulating factor (GM-CSF) (20 ng/ml, Peprotech, Rocky Hills, NJ, USA) for 6 days of culturing, as described (59). Cells were routinely verified to be >90% F4/80^+^. For activation experiments, cultured BMDMs were seeded at 2 × 10^5^ cells per well in 200 μl of standard RPMI-1640 medium and pretreated with hAAT at 0.5 or 2.5 mg/ml, as indicated, S-NO-AAT at 2.5 mg/ml or GSNO (Chem-Impex International, Wood Dale, IL, USA) at 27.5 μM. After 24 h, cells were carefully washed with PBS and activated with recombinant IFNγ (10 ng/ml, R&D Systems, Minneapolis, MN, USA) and IL-1β (10 ng/ml, Prospec, East Brunswick, NJ, USA), with or without coculturing with RMA cells at a ratio of 1:2 (RMA:BMDM) for 24 h. IL-6 release to the supernatant was quantified with specific ELISA (Biolegend, San Diego, CA, USA). Lactate dehydrogenase (LDH) release to the supernatant was quantified with a Non-Radiological Cytotoxicity Kit (Promega, Madison, WI, USA). Nitric oxide release to the supernatant was evaluated by Griess kit (Promega) according to the manufacturer’s instructions.

### Tumor Inoculation Models

In the B16-F10 primary tumor model, hAAT^+/+^ and C57BL/6 were untreated prior to inoculation of B16-F10 cells (5 × 10^5^ cells per animal, i.f.p.) or after inoculation. For *in vivo* transfection of hAAT, mice underwent hydrodynamic injections (HDI) of pEF-hAAT plasmid (100 μg per animal, i.v.), as described elsewhere (60). Control animals received the same volume of PBS, and serum samples were obtained routinely. Circulating hAAT levels were quantified with specific ELISA (Immunology Consultants Laboratory, Portland, OR, USA). Tumor length and width were measured with standard calipers. For examination of IRP effects in the B16-F10 primary tumor model, tumor-bearing limbs were subjected to unilateral ischemia by the application of a tourniquet for 3 h. Animals were sacrificed 2 h after removal of tourniquets, and total foot tissue from the immediate inoculation site was excised and dissociated by mechanical disruption with the gentleMACS Dissociator and C tubes (Miltenyi Biotec, Cologne, Germany). Purified RNA was then utilized for gene transcription quantification.

In the B16-F10 lung metastasis model, mice were treated with hAAT (2 mg per animal) or PBS 1 day prior to tumor cell inoculation and every third day thereafter. Mice subsequently underwent B16-F10 inoculation (1 × 10^6^ cells per animal, i.v.) and were sacrificed after 17 days. Peripheral blood was perfused, and lungs were fixed by perfusion of 4% paraformaldehyde prior to production of paraffin blocks. Metastatic foci in the lungs were subsequently counted by microsurgery and by identification of foci in H&E slides.

In the RMA/RMA-S and RMA/B16-F10 tumor cell clearance models, mice were treated with hAAT (2 mg per animal) or PBS 1 day prior to tumor cell inoculation. Inoculated cells were stained differentially with lipophilic tracers (RMA stained with DiL, RMA-S, and B16-F10 with DiD) (Thermo-Fisher, Waltham, MA, USA), mixed at a 1:1 ratio, and inoculated (1 × 10^7^ total cells per animal, i.v.*)*. Animals were sacrificed after 5 h, lungs were removed and dissociated, and lung cells were analyzed with flow cytometry.

In the RMA/RMA-S tumor model, mice were treated with hAAT (2 mg per animal) or PBS 1 day prior to tumor cell inoculation and every third day thereafter. Inoculation of RMA and RMA-S (1 × 10^5^ cells per animal, s.c.) was conducted either simultaneously to opposing flanks or separately to the right flank 24 h later, and tumor length and width were measured with standard calipers. Depletion of CD8^+^ T cells was conducted as described previously (61), and depletion outcomes were routinely verified to be the absence of >95% of CD8^+^ T cells compared to non-ablated mice. In certain experiments, RMA tumors were dissociated, and total tumor RNA was purified by mechanical disruption with the gentleMACS Dissociator and C tubes (Miltenyi Biotec) and utilized for gene transcription quantification.

### Primary T Cell Separation and Activation Assays

CD8^+^ T cells were purified from spleens and RMA tumors of tumor-bearing mice 13 days after inoculation of tumors by magnetic purification kit (Stemcell Technologies, Vancouver, BC, Canada) as per the manufacturer’s instructions. Cells were then incubated in 10% RPMI-1640 growth medium (Biological Industries) and cocultured with RMA cells (1:1 ratio) for 24 h. Cells were then stained for relevant intracellular markers, and TNFα release to the medium was quantified with specific ELISA (Biolegend, San Diego, CA, USA).

### Immunofluorescence

Immunofluorescent (IF) staining was performed with tissues fixed with 10% formaldehyde, dehydrated with ethanol, cleared in xylene, and embedded in paraffin. Tissues were sectioned with a microtome and 4 μm sections and stained with H&E or subjected to IF staining. For IF staining, PE-conjugated α-CD45 antibody was used at 1/20 dilution overnight (EM-05, Exbio, Prague, Czech Republic). Nuclei were stained with DAPI at 1/1,000 dilution (Sigma-Aldrich). Cells were examined using a 405-nm excitation laser line for DAPI and a 496-nm laser line for PE. Microscopy was performed using an Olympus Fluoview FV1000 confocal microscope.

### Flow Cytometry

In all experiments, 1 × 10^6^ cells were suspended in flow cytometry buffer consisting of PBS (Biological Industries) with 1% bovine serum albumin (BSA, Biological industries), 0.1% sodium azide, and 2 mM EDTA (Sigma-Aldrich). Fc blocking was conducted with α-CD16/32 antibody (Biolegend) for 20 min at 4°C. Staining was performed for 20 min at 4°C with the following antibodies: α-CD11c-APC (N418), α-MHC class II-PE (M5/114.15), α-F4/80-APC (BM80), α-TLR4-PE (MTS510), α-CD40-PE-Cy7 (3/23), α-CD86-PE (GL-1), α-CD8-PE-Cy7 (53-6.7), and α-CD11b-Pacific Blue (M1/70), all from Biolegend. Intracellular staining was conducted by treatment of target cells with Monensin solution (Biolegend) diluted 1/1,000 in standard RPMI-1640 growth medium for 5 h, followed by washing, standard Fc receptor blocking, surface antigen staining and fixation with Fixation Buffer (Biolegend) for 20 min. Cells were subsequently washed with Intracellular Staining Permeabilization Wash Buffer (Biolegend), and intracellular antigens were stained with the following antibodies: α-TNFα-Pacific Blue (MP6-XT22), α-IL-10-APC (JES5-16E3), α-IFNγ-AlexaFluor 647 (XMG1.2), and α-perforin-PE (BioOMAK-d) (all from Biolegend). For 7-AAD cellular viability assay, cultured cells were washed and introduced 7-AAD (Biolegend) diluted 1/200 in flow cytometry buffer. Cells were read in a flow cytometer within 10 min of exposure to 7-AAD reagent. Samples were read using BD Canto II (BD Biosciences, Singapore), and data were analyzed by FLOWJO 7.6.3 software (Flowjo, LLC Data Analysis Software, Ashland, OR, USA).

### Real-time PCR Assay

Total RNA extracted from surgically removed tumor tissues was purified with total RNA purification kit (Norgen Biotek Corp., Thorold, ON, Canada). After quantification of RNA by NanoDrop spectrophotometer (ND-1000, NanoDrop Technologies, Wilmington, DE, USA), reverse transcription of RNA to cDNA was performed using qScript™ cDNA synthesis kit (Quanta BioSciences, Gaithersburg, MD, USA) and 1 μg of template RNA and a blend of random hexamers and oligo (dT) primers. Quantification of gene transcription was performed with the StepOnePlus Real-Time PCR system and the Fast SYBR Green Master Mix (Applied Biosystems, Foster City, CA, USA). The following primers were used for PCR amplification: CD14: Fw 5′-CATTTGCATCCTCCTGGTTTCTGA Rev 5′-GAGTGAGTTTTCCCCTTCCGTGTG, IFNγ: Fw 5′-GTCATTGAAAGCCTAGAAAGTC Rev 5′-GTTGTTGACCTCAAACTTGG, GAPDH: Fw 5′-TCAACAGCAACTCCCACTCTTCCA Rev 5′-ACCCTGTTGCTGTAGCCGTATTCA, VEGF: Fw 5′-CAGGCTGCTGTAACGATGAA Rev 5′-AATGCTTTCTCCGCTCTGAA, α-SMA: Fw 5′-AGCACAATACCAGTTGTACG Rev 5′-ATAGAACACGGCATCATCAC, IL-1Ra: Fw 5′-CACCGGAAGAGCCCCTTATAG Rev 5′-TGCAAAAGTTGTTCCTCAGGC, IL-12p40: Fw 5′-TGGCGAACCTGGATGGTTT Rev 5′-GGGAGAGTCGGTTGCTTACC, and IL-10: Fw 5′-GCTCTTACTGACTGGCATGAG Rev 5′-CGCAGCTCTAGGAGCATGTG.

### Production of S-NO-AAT

Human α1-antitrypsin (20 mg/ml or 450 μM) was reduced by incubation with 50 mM DTT for 10 min at 37°C. Excess reducing agent was removed by gel filtration using Sephadex G-25 columns (GE Healthcare) equilibrated in the nitrosylation buffer (25 mM HEPES pH 7.4, 0.1 mM EDTA, 0.2 mM diethylenetriaminepentaacetate, 10 μM neocuproine, and 100 mM NaCl). Reduced hAAT was then incubated for 30 min with 1,000 μM diethylamine NON-Oate (Cayman Chemical, MI, USA) followed by adding additional 500 μM diethylamine NON-Oate for more 30 min at 37°C. After excess NO donor was removed by Sephadex G-25 chromatography, the S-nitrosylation efficiency was calculated by measuring protein concentration and number of nitrosylated thiols. Protein concentration was determined using Bicinchoninic acide (BCA) protein assay kit (Santa Cruz) with hAAT in known concentrations as a calibration curve. hAAT’s nitrosylated thiols quantification was carried out by Saville–Griess assay (62). Briefly, S-nitrosylated human AAT (S-NO-hAAT) was incubated in assay buffer (1% sulfanilamide, 0.1% *N*-(1-naphthyl)ethylenediamine dehydrochloride, 1% HCl) in the absence or presence of 1 mM HgCl_2_ for 30 min, and absorbance readings were measured at 540 nM by ELISA plate reader. S-nitrosoglutathione (GSNO) treated identically served as a standard curve, and mercury-dependent absorbance was converted to SNO concentrations. hAAT’s nitrosylation efficiencies were more than 50%. After production, S-NO-hAAT was aliquted into dark tubes and stored at −80°C for a single thaw. Saville–Griess assay was also used for S-NO-hAAT transnitrosylation; after treating peritoneal macrophages with 100 mM *N*-ethylmaleimide (NEM) or PBS for 15 min at RT, they were introduced to S-NO-hAAT. Medium supernatant samples were taken in indicated time points, and the S-nitrosylated protein was measured by Saville–Griess assay.

### Statistical Analysis

Statistical analysis results are expressed as mean ± SEM. Unpaired two-tailed Student’s *t*-test was used to assess the differences between treated groups and control. A *p*-value of ≤0.05 was considered significant.

## Results

### Comparison between Constitutively Elevated hAAT and Plasmid-Derived Circulating hAAT in a B16-F10 Footpad Tumor Model

**Figure 1:**
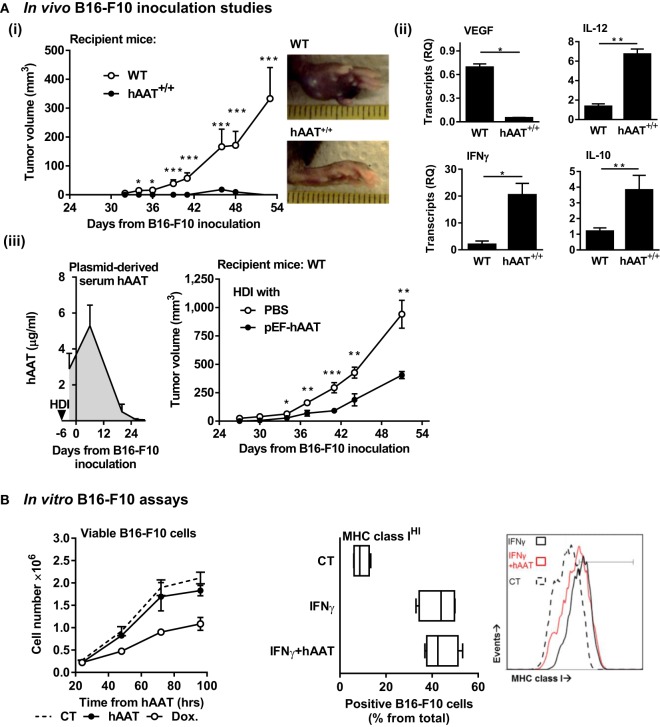
**Plasma and plasmid-derived hAAT inhibit *in vivo* development of primary B16-F10 tumors**. **(A)** (i) hAAT^+/+^ and C57BL/6 mice (*n* = 10 and 11 per group, respectively) were inoculated i.f.p. with B16-F10 melanoma cells (5 × 10^4^ cells per mouse). Footpad tumor size and representative day-38 footpad tumor photomicrographs. (ii) On day 21 after inoculation of C57BL/6 and hAAT^+/+^ mice, total tumor RNA was purified, and transcription levels of designated genes were determined. Mean ± SEM, *p < 0.05, **p < 0.01, ***p < 0.001. Unpaired two-tailed Student’s *t*-test was employed to assess differences between groups at each time point. (iii) C57BL/6 mice (*n* = 8 per group) were introduced PBS or pEF-hAAT by hydrodynamic tail–vein injection. Six days later, mice were inoculated with B16-F10 cells i.f.p. (5 × 10^4^ cells per mouse). Circulating serum levels of hAAT and tumor size follow-up. Triangle, time of *in vivo* plasmid introduction. **(B)** *Left*: B16-F10 cells (3 × 10^5^ cells in triplicate) were pretreated *in vitro* with hAAT (0.5 mg/ml) or doxorubicin (60 μM), and cell numbers were counted at indicated time points. Mean ± SEM. *Right*: B16-F10 cells (3 × 10^5^ cells in triplicate) were pretreated with hAAT (0.5 mg/ml) for 24 h and then stimulated with IFNγ (10 ng/ml) for 24 h. Cells were then stained for MHC class I and analyzed by flow cytometry. Mean ± SEM.

In order to exclude the possibility that tumors might thrive in conditions of elevated levels of circulating hAAT, the initial assessment of the outcomes of hAAT treatment on development of B16-F10 primary tumors was performed. Tumor development after i.f.p. inoculation of B16-F10 cells was tested in WT mice and in mice that constitutively overexpress hAAT (hAAT^+/+^) (56). Importantly, hAAT^+/+^ mice were used in multiple experimental settings by our group as well as others and constitutively display similar steady-state immunological background (9, 63, 64). As shown in Figure 1A (i), while WT mice displayed measurable tumors within the overall expected time frame (~30 days), hAAT^+/+^ mice developed discernable tumors at a delayed time point (~42 days), and these tumors failed to thrive.

The significantly reduced development of tumors observed in hAAT^+/+^ mice required that we investigate the *in vivo* antitumor properties of hAAT in this model. Transcription levels of pro-tumor and antitumor cytokines that are primarily leukocyte-derived in the tumor environment were therefore evaluated. As shown in Figure 1A (ii), hAAT^+/+^ mice displayed significantly reduced VEGF transcript levels and elevated IL-12p40 and IFNγ transcript levels, as compared to WT animals.

Tumor development was further evaluated using an *in vivo* transfection model. A hAAT-expressing plasmid (pEF-hAAT) was introduced into WT mice utilizing the HDI model [Figure 1A (iii)]. Control WT mice underwent HDI with identical volumes of PBS. Mice were then inoculated i.f.p. with B16-F10 cells, and tumor size was continuously examined. As shown, mice expressing plasmid-derived hAAT displayed micrograms of circulating hAAT, albeit for a limited period of time (*left*). Primary tumor development in these mice was significantly delayed and diminished compared to parallel time points in control mice (*right*). In contrast to *in vivo* models examined, *in vitro* tumor cell proliferation was not inhibited by hAAT (Figure 1B *left*), and tumor cells displayed intact inducible MHC class I expression when treated with hAAT (Figure 1B *right*).

### The Interface between Tissue-Infiltrating B16-F10 Cells and Local Leukocyte Responses: Metastatic Spread

**Figure 2:**
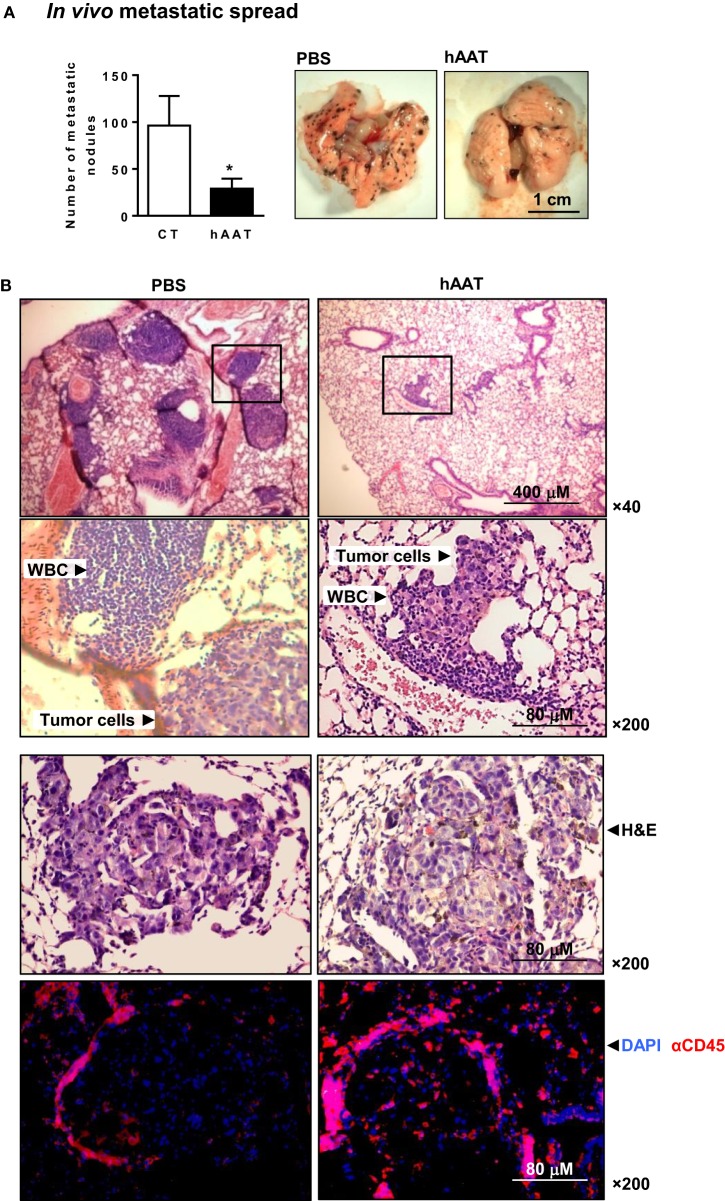
**Diminished metastatic spread of B16-F10 cells in hAAT-treated mice**. **(A)** C57BL/6 mice (*n* = 8 per group) were pretreated with hAAT (2 mg per animal) 24 h prior to inoculation with B16-F10 cells (1 × 106 cells per animal) and every third day afterward. Animals were sacrificed after 17 days. Lung metastatic nodules were counted by microsurgery. Mean ± SEM, *p < 0.05. Unpaired two-tailed Student’s *t*-test was employed to assess differences between groups. **(B)** Histological sections were taken 17 days post-inoculation. Representative photomicrographs of lung samples stained with H&E or immunofluorescently stained with DAPI and α-CD45.

In order to examine the outcomes of hAAT treatment on peripheral tumor cell infiltration, i.v. inoculation of B16-F10 cells was conducted. Lung metastatic nodules were counted after 17 days (Figure 2A). As shown, metastatic spread was significantly reduced in mice treated with hAAT. During histological examination of explanted lungs (Figure 2B), metastatic nodules were identified and PBS-treated animals tumor foci that were spatially separate from leukocytic infiltrates. In contrast, hAAT-treated mice displayed a lower degree of leukocyte infiltration, but immunocytes were interspersed between tumor cells, as demonstrated by the higher number of metastatic node-infiltrating CD45^+^ leukocytes in hAAT-treated mice. At large, it appears that hAAT treatment resulted in reduction of metastatic *burden*, without a significant reduction in leukocyte recruitment to the vicinity of tumor foci.

### NK Cell- and T Cell-Sensitive Tumor Models

The influence of hAAT therapy over *in vivo* tumor cell clearance without prior exposure to tumor cells was examined. Animals were treated with hAAT and 24 h later inoculated i.v. with a 1:1 ratio of NK cell-sensitive RMA-S lymphoma cells (65) and NK cell-*insensitive* RMA T cell lymphoma cells (66), both differentially stained with lipophilic dyes. After 5 h, animals were sacrificed, and the presence of both cell types in the lungs was examined by flow cytometry. As shown in Figure S1A in Supplementary Material, the ratio of the two cell types at the end of 5 h was uniform between PBS- and hAAT-treated mice (approximately eight times as many NK-insensitive RMA cells than NK-sensitive RMA-S cells). When a similar experimental set-up was performed with RMA and B16-F10 cells, PBS- and hAAT-treated mice again displayed similar ratios of NK-insensitive RMA cells and NK-sensitive B16-F10 cells (Figure S1B in Supplementary Material).

**Figure 3:**
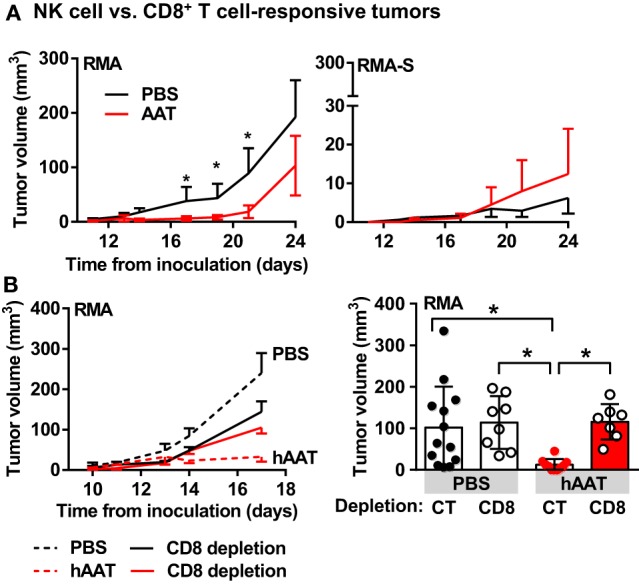
**hAAT-treated mice display inhibited development of RMA, but not RMA-S tumors**. **(A)** C57BL/6 mice (*n* = 8 per group) were pretreated with hAAT (2 mg per animal, i.p.) 24 h prior to simultaneous inoculation with RMA cells to the right flank and RMA-S cells to the left flank (1 × 10^5^ cells per animal, s.c.); hAAT was administered every third day afterward, and tumor sizes were determined routinely. **(B)** *In vivo* RMA inoculations (*n* = 6 per group) were combined with depletion of CD8^+^ T cells, and tumor size was measured routinely. Graphs represent linear development of tumors as well as full scatter graph at day 17 post-inoculation. Mean ± SEM, *p < 0.05. Unpaired two-tailed Student’s *t*-test was employed to assess differences between groups.

The two main lymphocytic compartments responsible for effective *in vivo* clearance of tumor cells are NK cells (67) and CD8^+^ T cells (68). In order to specifically determine the effects of hAAT on NK cell and cytotoxic CD8^+^ T cell activity in the context of *in vivo* tumor development, mice were simultaneously inoculated s.c. with CD8^+^ T cell-sensitive, NK cell-insensitive RMA cells to the *right* flank (69, 70), and with CD8^+^ T cell-insensitive, NK cell-*sensitive* RMA-S cells to the *left* flank (Figure 3A), and tumor size was determined throughout a 24-day follow-up. As shown, hAAT-treated mice displayed significant inhibition of RMA tumor development. NK cell-*sensitive* RMA-S tumors were slow to grow and unaffected by hAAT therapy. Importantly, when mice were subjected to identical hAAT treatment regimens and inoculated separately with RMA or RMA-S cells, development patterns of both types of tumors were similar to those observed in mice inoculated with both cell lines simultaneously (Figure S2 in Supplementary Material).

The involvement of CD8^+^ T cells in tumor growth inhibition by hAAT was then challenged. RMA tumor development was examined in mice depleted of CD8^+^ T cells with or without hAAT treatment, as followed over 17 days. As shown in Figure 3B *left (follow-up)* and *right (day 17)*, hAAT-mediated inhibition of RMA tumor growth was compromised by CD8^+^ T cell depletion. These outcomes suggest that the observed inhibition is dependent on CD8^+^ T cell activity, and that NK cells retain intact antitumor capacity during *in vivo* hAAT treatment. As hAAT lacks direct influence on T cell responses (4, 71), an indirect avenue of modulation by way of innate immune cell-alteration was next considered.

### Tumor-Associated Innate Leukocyte Populations in hAAT-Treated Mice

**Figure 4:**
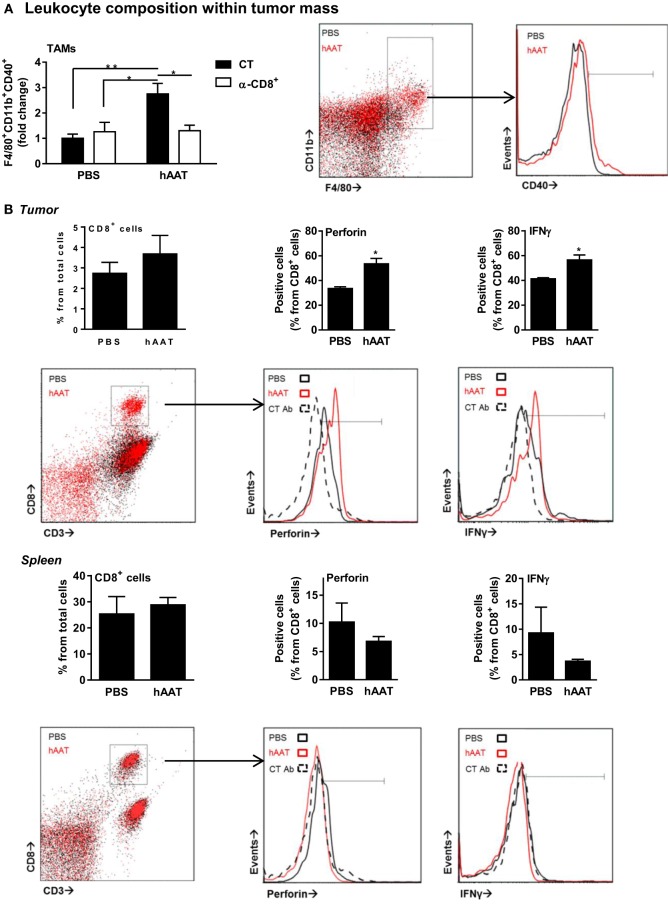

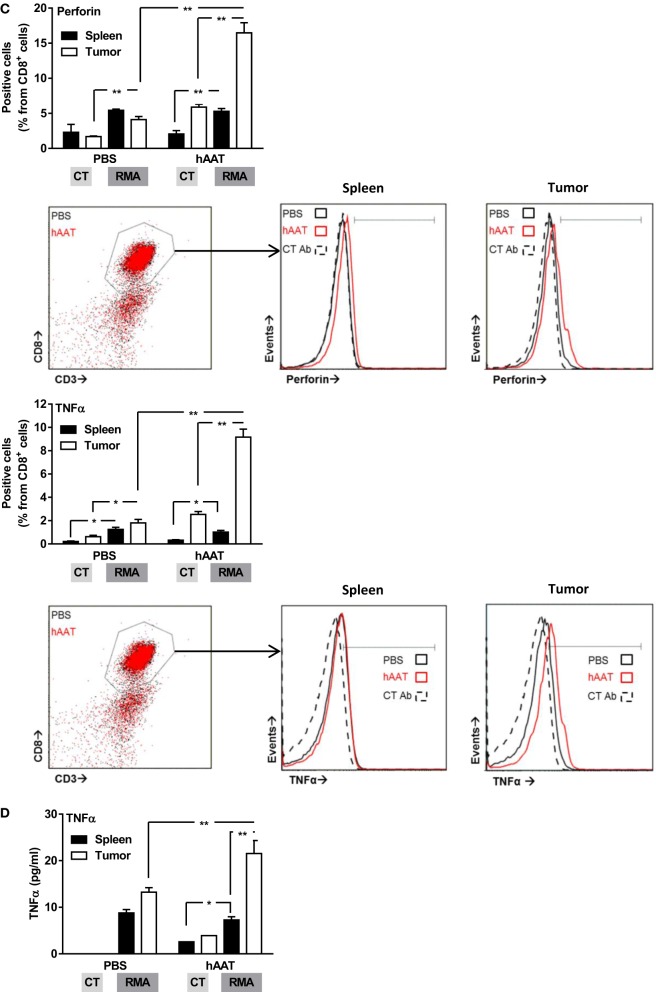
**Increased activation marker expression by RMA tumor-infiltrating leukocytes in hAAT-treated mice**. **(A)** PBS- and hAAT-treated RMA tumor-bearing mice (*n* = 4 per group) were sacrificed 13 days after RMA cell inoculation (1 × 10^5^ cells per animal, s.c.). Tumors were dissociated, and cell populations were stained for indicated markers. **(B)** Tumors and spleens of RMA tumor-bearing animals were dissociated, and T cell populations were stained and analyzed by flow cytometry. **(C,D)** Purified splenic or tumor-infiltrating CD8^+^ T cells (1 × 10^5^ cells per well in triplicate) were incubated for 24 h with or without cultured RMA cells (CT:RMA, respectively) (1:1 ratio). Cells were then stained for **(C)** intracellular perforin and TNFα, and **(D)** release of TNFα to the supernatant was quantified. Mean ± SEM, *p < 0.05, **p < 0.01. Unpaired two-tailed Student’s *t*-test was employed to assess differences between groups.

The influence of hAAT on innate leukocyte interaction with tumors was examined in tumor-bearing mice. Solid RMA tumors were dissociated in animals treated with hAAT, depleted of CD8^+^ T cells, or a combination of both, and tumor-infiltrating leukocyte populations were depicted. As shown in Figure 4A *solid bars*, a significantly elevated percentage of tumor-infiltrating F4/80^+^CD11b^+^CD40^+^ cells was detected, agreeing with greater prevalence of M1-like TAMs in hAAT-treated mice (72–74). Importantly, TAMs present in hAAT-treated mice that were depleted of CD8^+^ T cells displayed a profile similar to PBS-treated mice (Figure 4A *empty bars*). CD11c^+^ DCs detected in RMA tumors of hAAT-treated mice contained significantly greater percentages of CD40^+^ DCs (Figure S3 in Supplementary Material), similar to F4/80^+^CD11b^+^ TAMs. Percentages of CD11c^+^CD40^+^ DCs in hAAT-treated mice that were depleted of CD8^+^ were similar to percentages detected in PBS-treated mice (Figure S2 in Supplementary Material), again conforming to observations made for F4/80^+^CD11b^+^ TAMs.

The profile of cytotoxic CD8^+^ T cells in hAAT-treated mice was tested *in vivo*. Primary RMA tumors were dissociated, and tumor-infiltrating T cell populations were stained for intracellular perforin and IFNγ. As shown in Figure 4B *top*, while total tumor-associated CD8^+^ T cell percentages were similar between PBS- and hAAT-treated mice, tumor-infiltrating CD8^+^ T cells in hAAT-treated mice were found to express significantly greater levels of perforin and IFNγ. Splenocytes from tumor-bearing mice (Figure 4B *bottom*) appear to have been unaffected by the treatment, with a possible marginal trend of the spleen toward being depleted from perforin/IFNγ-expressing T cells. These results suggest that CD8^+^ T cells in hAAT-treated mice may be more prone to antitumor cytotoxic functions, in the examined models.

### *In Vitro* Activation of CD8^+^ T Cells from Tumor-Bearing Mice

Magnetically purified tumor and spleen CD8^+^ T cells were cocultured with RMA target cells for 24 h. Cells were stained for intracellular perforin, TNFα, and (*not shown*) IFNγ. As depicted in Figure 4C, perforin and TNFα staining levels were significantly greater in all examined CD8^+^ T cell populations when RMA target cells were present, and while perforin and TNFα levels were similar for spleen CD8^+^ T cells from PBS and hAAT-treated mice (*solid bars*), tumor CD8^+^ T cells from hAAT-treated mice expressed significantly greater perforin and TNFα levels than either spleen or tumor CD8^+^ T cells from PBS-treated cells (*empty bars*). Intracellular IFNγ staining was not significantly elevated by the presence of RMA target cells and was similar in all examined CD8^+^ T cell populations (*not shown*). Release of TNFα to the supernatant was also examined (Figure 4D), induced by coculture of RMA cells with all examined CD8^+^ T cell populations. As shown, TNFα levels were consistent between spleen CD8^+^ T cell–RMA cocultures, while CD8^+^ T cell originating from tumors of hAAT-treated mice released significantly higher levels of TNFα (*second solid bar from the right*), thus conforming to the intracellular staining shown for TNFα (Figure 4C).

### Unique Tumor Inflammatory Gene Expression under hAAT Treatment

**Figure 5:**
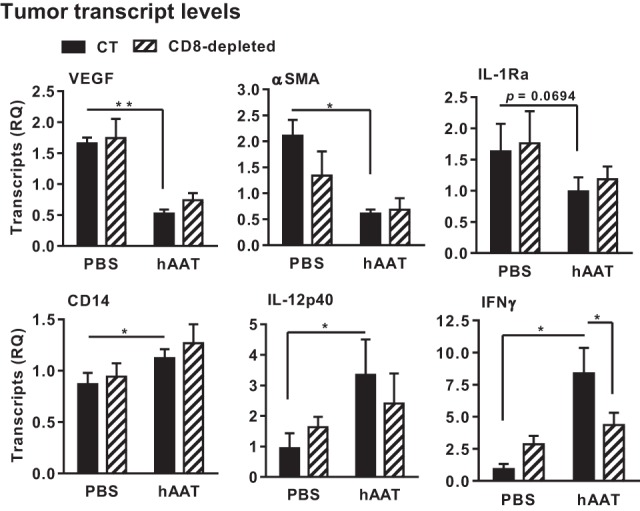
***In vivo* hAAT treatment induces pro-inflammatory gene expression profile in RMA tumor mass**. PBS- and hAAT-treated RMA tumor-bearing mice (*n* = 3 per group) were sacrificed 17 days after RMA cell inoculation (1 × 10^5^ cells per animal, s.c.). Tumors were excised and processed for real-time QPCR gene expression analysis for indicated genes. Mean ± SEM, *p < 0.05. Unpaired two-tailed Student’s *t*-test was employed to assess differences between groups.

In order to examine inflammation-associated gene expression in the tumor, total RMA tumor tissue RNA was purified, and a series of real-time QPCR gene transcription assays was performed. Animal groups were comprised of hAAT-treated CD8^+^ T cell-depleted groups. As shown in Figure 5, a significant reduction in VEGF and α-SMA transcript levels was detected in hAAT-treated mice regardless of CD8^+^ T cell depletion. Unexpectedly, transcription of IL-1Ra was also reduced in hAAT-treated mice, although to lesser extent. IL-12p40 and CD14 transcripts were conversely found to rise in hAAT-treated mice, and likewise unaffected by CD8^+^ T cell depletion. Foxp3 and IL-10 transcription levels were similar in all groups; IFNγ transcription was found to be higher in hAAT-treated mice, but levels were similar to PBS-treated mice when hAAT administration was combined with CD8^+^ T cell depletion.

These results demonstrate that hAAT treatment alters inflammation-associated gene expression in tumors; angiogenesis and M2-like macrophage-associated gene transcription appears to be *decreased*, while M1-like macrophage gene transcription exhibits a tendency to rise regardless of the presence of cytotoxic CD8^+^ T cells (30).

Importantly, IFNγ transcription was significantly *increased* by hAAT treatment *only* when CD8^+^ T cells were intact.

### Effect of hAAT on Macrophage–RMA Cocultures

**Figure 6:**
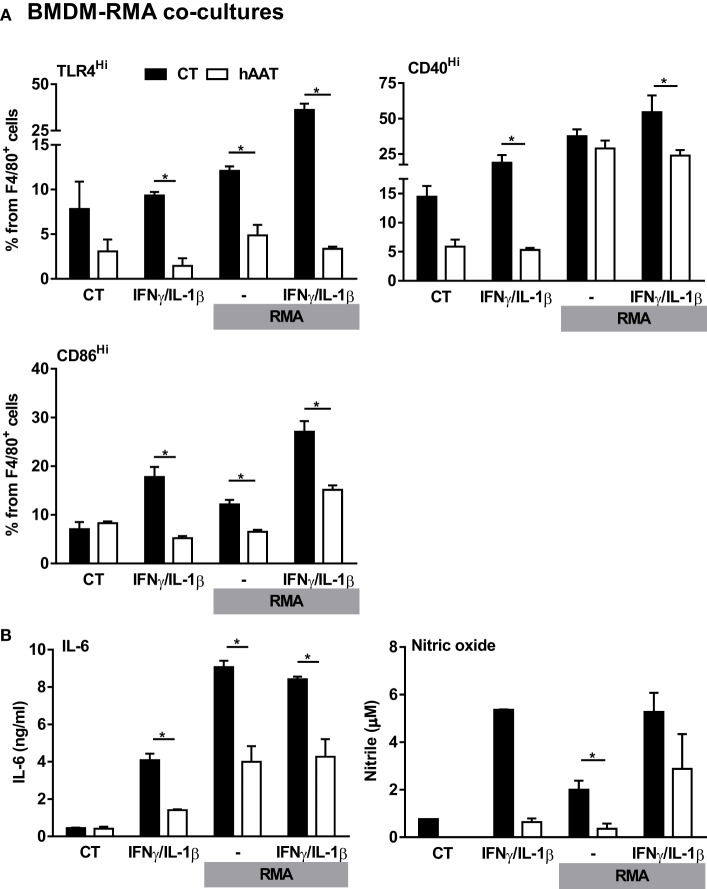
**Unmodified hAAT exerts anti-inflammatory effect on BMDM–RMA cocultures**. BMDMs (1 × 10^6^ cells per well in triplicate) were pretreated with hAAT (0.5 mg/ml); after 24 h BMDMs were cocultured with RMA cells (2.5 × 10^5^ cells per well) with or without stimulation with IFNγ and IL-1β (both at 10 ng/ml). RMA cells were diluted 1:4 after 48 h, and after 72 h **(A)** cells were washed and stained for flow cytometric analysis, and **(B)** supernatants were examined for IL-6 and NO levels. Mean ± SEM, *p < 0.05. Unpaired two-tailed Student’s *t*-test was employed to assess differences between groups.

Thus far, evidence has been gained regarding the effects of hAAT on *in vivo* tumor immunology with the particular observation of M1-like macrophage polarization and associated induction of CD8^+^ T cell antitumor cytotoxicity, an intriguing opposite trend to the overall knowledgebase regarding hAAT and inflammation (4, 5, 9, 14, 17, 60). In order to clarify the mechanism by which hAAT may act to affect tumor-infiltrating macrophages in such a context-dependent manner, BMDMs were pretreated with hAAT and then cocultured with RMA tumor cells, with or without stimulation with recombinant IFNγ and IL-1β. After 72 h, BMDMs were stained and analyzed for M1-like macrophage markers, TLR4 and CD40 (Figure 6A). As shown, the response of stimulated BMDMs to RMA cells was effectively inhibited by pretreatment with hAAT. Consistent with this finding, IL-6 and NO release to the supernatant were diminished by hAAT (Figure 6B).

### Nitrosylated hAAT Facilitates M1-Like BMDM Stimulation

**Figure 7:**
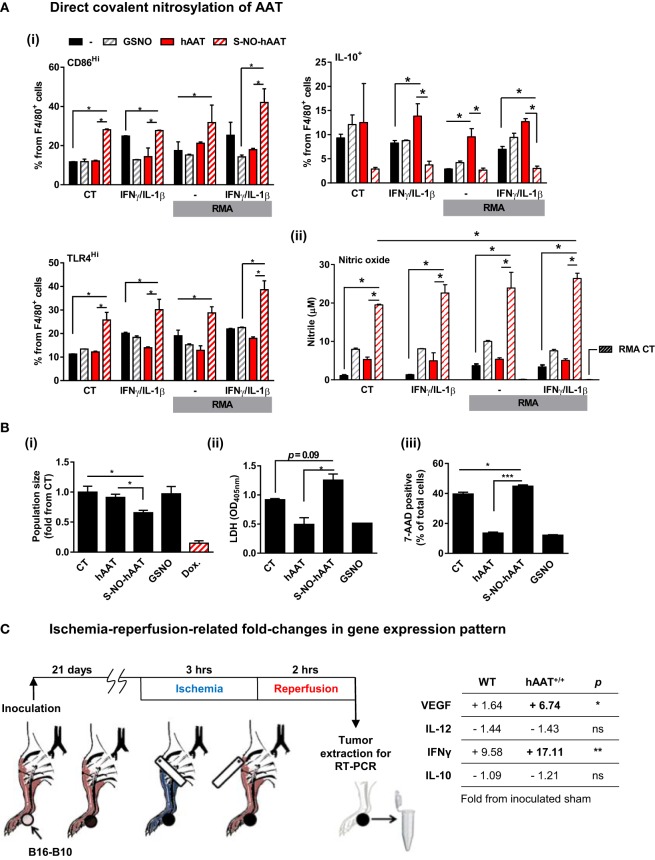
**Evidence for context-specific activities of hAAT**. **(A,B)** S-NO-AAT induces pro-inflammatory effects on BMDM stimulation and tumor cell viability. **(A)** BMDMs (1 × 10^6^ cells per well in triplicate) were pretreated with hAAT (2.5 mg/ml), S-NO-hAAT (2.5 mg/ml), or GSNO (27.5 μM) and after 24 h were cocultured with RMA cells (2.5 × 10^5^ cells per well) with or without stimulation with IFNγ and IL-1β (both at 10 ng/ml). RMA cells were diluted 1:4 after 48 h, and after 72 h (i) cells were washed and stained for flow cytometric analysis, and (ii) supernatants were analyzed for NO levels. Mean ± SEM, *p < 0.05. Unpaired two-tailed Student’s *t*-test was employed to assess differences between groups. **(B)** RMA cells were seeded at 1 × 10^5^ cells per well and treated with hAAT, S-NO-hAAT (both at 2.5 mg/ml), GSNO (27.5 μM), or doxorubicin (60 μM). (i) Cells were stained with trypan blue, and the population of viable cells was determined after 24 h. (ii) LDH release after 48 h. (iii) 7-AAD staining. Mean ± SEM, *p < 0.05, ***p < 0.001. Unpaired two-tailed Student’s *t*-test was employed to assess differences between groups. **(C)** Effect of ischemia–reperfusion on intra-tumor gene expression profile during hAAT treatment. C57BL/6 mice (*n* = 3 per group) were pretreated with hAAT (2 mg per animal, i.p.) 24 h prior to inoculation of B16-F10 melanoma cells (5 × 10^4^ cells per mouse, i.f.p.). Mice were subjected to limb ischemia–reperfusion (3 h ischemia; 2 h reperfusion) on day 21 post-inoculation. Mice were then sacrificed, and total foot tissue surrounding the inoculation site was excised and processed for real-time QPCR gene expression analysis for indicated genes. Results are presented as fold-change from inoculated sham-operated mice. *p < 0.05, **p < 0.01 between hAAT^+/+^ and WT groups. Unpaired two-tailed Student’s *t*-test was employed to assess differences between groups.

Considering that hAAT is broadly regarded as an anti-inflammatory agent (1), the possibility that local covalent modifications might alter its function in a context-dependent manner was examined. Treatment of BMDMs with cysteine-nitrosylated hAAT (S-NO-hAAT) revealed a unique pattern (Figure 7A). BMDMs were stimulated with IFNγ and IL-1β and treated by either hAAT, S-NO-hAAT, or GSNO (at equal molar ratios of NO to S-NO-AAT) before being exposed to RMA cells. The response of BMDMs was depicted by cell-specific staining for surface CD86 and intracellular IL-10. As shown in Figure 7A (i), while naïve hAAT (*red*) reduced BMDM expression of CD86 and elevated the expression of IL-10, treatment with S-NO-hAAT largely reversed this phenotype; the presence of GSNO had no discernable effect. Treatment of cell cultures with S-NO-hAAT also significantly raised NO release, as depicted in Figure 7A (ii); importantly, NO levels in S-NO-hAAT-treated cultures were significantly greater than NO levels in GSNO-treated wells (*gray*), suggesting that the rise in NO release stems from *increased activation* of BMDMs rather than the molecular introduction of free NO.

The influence that S-NO-hAAT may have on tumor cell survival was next addressed. Tumor cells (RMA) were treated *in vitro* with S-NO-hAAT (Figure 7B). Control treatments consisted of unmodified hAAT, nitrosylated glutathione (GSNO) at an equimolar concentration, and doxorubicin (Dox.) as a positive control for tumor cell death. Cells were seeded and counted after 24 h. As shown in Figure 7B (i), wells cultured with S-NO-hAAT were found to contain significantly lower cell numbers at 24 h compared to control or wells cultured with either hAAT or GSNO. After 48 h, supernatants were collected for assessing LDH release, and cell death events were recorded by staining with 7-AAD. As shown in Figure 7B (ii) and (iii), supernatant LDH levels were significantly higher in wells added S-NO-hAAT, while staining with 7-AAD yielded significantly greater staining percentages in cells treated with S-NO-hAAT. These results suggest that tumor cell viability may be inhibited to a certain extent by the presence of S-NO-hAAT; this effect is not observed when naïve hAAT or GSNO are added. Interestingly, naïve hAAT appeared to have diminished the readout of cell death in RMA cells but did not alter the total amount of tumor cells. Thus, if *in situ* local tumor conditions are rich in NO, they may modify hAAT to a form, which facilitates tumor cell susceptibility.

### Ischemia–Reperfusion of Tumor Tissue Differentially Alters Cytokine Transcription in WT and hAAT^+/+^ Transgenic Mice

Temporary oxygen deprivation followed by arterial blood irrigation induces numerous changes in gene transcription levels (75). Using B16-F10 tumor-bearing WT and hAAT^+/+^ mice, we performed IRP of tumor-bearing extremities (Figure 7C) and determined changes in VEGF, IL-12p40, IFNγ, and IL-10 transcription levels in the immediate inoculation site. As shown, tumors collected from WT animals that underwent IRP displayed a rise in VEGF and IFNγ transcription levels and a decline in IL-12p40 and IL-10 levels. While the same overall trend is readily observed in tumors from hAAT^+/+^ animals, there appears to be an even greater presence of VEGF and IFNγ (4.1- and 1.78-fold from IRP-treated tumors collected from WT mice, respectively).

## Discussion

The immunomodulatory effects of AAT in autoimmune and allogeneic immune responses have been thoroughly studied, consistently demonstrating amelioration of pathological processes and induction of long-lasting tolerance. AAT is known to promote leukocyte profiles associated with inflammatory resolution and tissue regeneration (1, 76). While tumors may theoretically benefit from an immunomodulatory environment rich in growth factors and with reduced lymphocytic activation, one must consider that numerous physical and immunological differences exist between autoantigen-driven immune models and tumor-related settings. Indeed, routine AAT treatment, as afforded to individuals with AAT deficiency (AATD) (77), has been commonplace for over three decades, and clinical evidence points to a reduction in bacterial infections in AAT-treated AATD individuals (76) as well as a lack of rise in tumor development rates. In fact, if untreated, individuals with AATD display *elevated* rates of lung cancer (78, 79), gastrointestinal cancer (80, 81), and liver cancer (82, 83).

The scarcity of knowledge regarding the involvement of AAT in tumor development and tumor immunology necessitates a careful examination of tumor development dynamics in immune-competent settings. Such an examination must elucidate the effects of various AAT administration regimens. Initial experiments performed in this study made use of a model in which B16-F10 melanoma cells were inoculated into hAAT^+/+^ transgenic mice that constitutively overexpress hAAT (56). We were surprised to find that B16-F10 tumors failed to thrive over extended periods of time. Since transgenic animal colonies are at risk of harboring discrepancies from their founders and crossbred background strains, the study was subsequently conducted in WT mice that were either exogenously injected with serum-purified clinical-grade hAAT or transfected *in vivo* with a hAAT-expressing liver-directed plasmid. The outcomes were indeed in line with tumor growth suppression in hAAT^+/+^ mice, and subsequent experiments were set forth to explore the cellular responders to hAAT therapy under these conditions.

*In vitro* treatment of B16-F10 cells with hAAT demonstrated that cellular viability is not directly reduced by hAAT, and that IFNγ-induced upregulation of MHC class I expression remains intact in its presence. A significant reduction in VEGF transcription and elevation in IL-12p40 and IFNγ transcription levels was detected in tumors from hAAT^+/+^ mice. These findings suggest the presence of an antitumor cytotoxic response rather than direct oncological stress. Moreover, in the *in vivo* hAAT-plasmid transfection model, hAAT-expressing animals had significantly delayed growth rates of B16-F10 tumors even though circulating hAAT levels subsided ~14 days after tumor inoculation. C57BL/6-background hAAT^+/+^ mice (56) have been studied in multiple immunological models, including EAE, ulcerative colitis, transplantation, vaccination, and bacterial infections (1). Steady-state leukocyte compartments are at large similar to WT mice in general population size and activation profile (9); differences are primarily observed in modified outcomes of immunological activation.

In order to further establish the mechanism of action by which AAT inhibits tumor development *in vivo*, we examined the metastatic spread of B16-F10 cells. Using hAAT-treated animals, we found that hAAT treatment significantly reduced the number of metastatic nodules in the lungs. Interestingly, in hAAT-treated mice, leukocyte infiltration toward the area of metastatic foci was intact and displayed greater staining levels of CD45^+^ cells, compared to vehicle-treated animals. Additionally, it was apparent that lymphocytic infiltration was submerged within the tumor mass, as opposed to the vehicle-treated group that displayed tumor masses forming discrete areas adjacent to non-invasive aggregates of mononuclear cells. hAAT thus appears to *permit* leukocyte extravasation given a strong enough inflammatory stimulus (e.g., establishment of metastatic cells).

Clearance of tumor cells may be executed by several immune cell types and in several discreet time spans. In hAAT-treated mice, survival rates of NK cell-sensitive RMA-S cells after i.v. inoculation were found to be comparable to those of NK cell-*insensitive* RMA cells, suggesting that *in vivo* hAAT administration allows NK cell clearance of tumor cells, at least in this said time frame. Similar results were obtained when the survivability of i.v.-*inoculated* B16-F10 cells was compared to that of RMA cells.

B16-F10 cells may be lysed by sensitized CD8^+^ T cells as well as NK cells. In order to characterize the leukocyte compartment primarily responsible for an increased antitumor immune response in hAAT-treated animals, we compared the progression of RMA (CD8^+^ T cell-sensitive, NK cell-insensitive) (70) and RMA-S (CD8^+^ T cell-insensitive, NK cell-sensitive) tumors (57) in direct inoculation experiments. While ablation of either NK cells or CD8^+^ T cells in B16-F10-inoculated animals is feasible, we employed the RMA/RMA-S inoculation model as these cells have been thoroughly studied in this context and reliably depict their distinct sensitivity to NK or CD8^+^ T cells. Here, while NK cell-sensitive tumors were unaffected by hAAT treatment, CD8^+^ T cell-sensitive tumors were significantly inhibited by hAAT treatment. These results suggest that, in contrast to observations collected from antigen-elicited immune settings, antitumor CD8^+^ T cell activity is in fact *amplified* by hAAT. Furthermore, CD8^+^ T cells proved to be indispensable for hAAT-induced inhibition of RMA tumors, based on a CD8^+^ T cell depletion experiment. Importantly, although NK cell activity did not appear to be elevated by hAAT treatment, it was not diminished either. These outcomes, as obtained by inoculation studies, suggest that NK cells or CD8^+^ T cell depletion studies should follow in order to fully elucidate the relevance of each cell subtype to the activity of AAT.

The characterization of tumor-infiltrating leukocytes yielded several interesting findings. For example, TAM expression of CD40 was significantly greater in hAAT-treated animals. Collectively, these observations correlate with the significantly elevated activation profile of tumor-infiltrating CD8^+^ T cells. Notably, such elevated CD8^+^ T cell activation was absent in the spleen of tumor-bearing animals, signifying the importance of *local*, rather than systemic, conditions exerted by hAAT treatment in the whole animal.

Analysis of intra-tumor cytokine transcription levels suggests that AAT administration promotes a pro-cytotoxic inflammatory profile inside the tumor, including elevated transcription levels of IL-12p40, CD14, and IFNγ, and reduced transcription of IL-1Ra, VEGF, and α-SMA. This unique composition of expressed genes supports the presence of a profound distinction between tumor-related activities of hAAT and benign conditions in its presence; islet grafts and endothelial cells were shown to express elevated levels of VEGF in the presence of hAAT (84), agreeing with the fact that the promoter for hAAT is hypoxia-responsive (85). Thus, the reduction in VEGF expression in the presence of hAAT suggests the presence of a distinguishing component that alters the transcriptome of hAAT-treated tumors and is independent of hypoxic conditions. Importantly, IFNγ transcription, but not IL-12p40 transcription, was affected by CD8^+^ T cell depletion; thus, hAAT appears to induce the upregulation of CD8^+^ T cell-driving factors, such as IL-12p40, in a manner independent of the presence of CD8^+^ T cells. Earlier observations have determined that IL-2-dependent activities of CD8^+^ T cells are not directly affected by hAAT; we therefore suggest that hAAT-induced changes in the intra-tumor inflammatory gene profile are primarily mediated by innate leukocytes, such as macrophages.

In order to further clarify the mechanism by which hAAT appears to induce greater antitumor activation of CD8^+^ T cells, future studies regarding hAAT may make use of tumor inoculation models in conjunction with suppression of inflammation. Specifically, sorting out of intra-tumor macrophages, as well as antibody-mediated depletion of specific leukocyte compartments other than T cells could be used in order to clarify the interdependency of hAAT-induced T cell activation and macrophages, as well as other innate immune populations.

Polarization of TAMs to a pro-inflammatory profile has been documented to facilitate antitumor responses (52, 86, 87). Our results suggest that under conditions of an evolving solid tumor, hAAT treatment induces elevated expression of CD40 by TAMs and thus the acquisition of pro-inflammatory properties. This favorable profile stands in contrast to the typical anti-inflammatory and tissue-regenerative activities of hAAT, which include elevated IL-10 levels and further emphasizes the possibility that serum hAAT is distinct from tumor environment hAAT. Indeed, hAAT treatment seems to evoke diametrically opposed outcomes between tumor immunology and autoimmune responses. An attempted explanation for such differences in activity must take the vastly different immune-related environments that exist in tumors and in benign antigenic responses into account.

Nitric oxide holds a central and complex role in tumor development. Reports suggest that NO may reduce tumor cell proliferation and induce tumor cell apoptosis (88) but may also facilitate immunosuppressive leukocytes and promote growth factor secretion (88). Importantly, NO activity may be conducted through covalent modification of proteins and specifically by the S-nitrosylation of a free cysteine residue (89, 90). Protein-bound non-gaseous NO groups may then be transferred between proteins, including membrane proteins (91, 92). Covalent modification of proteins grants a high degree of specificity to subsequent NO function, as opposed to unbound gaseous NO. Notably, hAAT has been shown to readily undergo S-nitrosylation on its single surface cysteine residue during inflammatory stimulation, forming S-NO-AAT (12).

Our results suggest that S-NO-AAT induces pro-inflammatory polarization in cultured BMDMs under stimulation with pro-inflammatory cytokines. Our findings also indicate that treatment of BMDMs with S-NO-AAT leads to greater release of NO to the supernatant, potentially creating a positive-feedback cycle around NO. Furthermore, exposure of RMA cells directly to S-NO-AAT resulted in significant reduction in tumor cell viability. Importantly, although treatment with S-NO-AAT reduced RMA cell viability, it did not alter the overall size of tumor cell population, suggesting that *in vivo* inhibition of tumor development by hAAT requires factors beyond high NO levels. While the concentration of S-NO-hAAT in the present study is extrapolated from reports on circulating AAT levels during acute phase responses, it is still of interest to explore local S-nitrosylation of naïve AAT inside the tumor in future studies.

Induction of AAT expression by hypoxic conditions may represent a compounding entity in the physiologic reasoning of the growth-inhibiting influence of hAAT. Unlike in the case of antigen-related immune settings, primary tumors are uniformly characterized by the presence of hypoxia. Such conditions may alter the function of local hAAT. Here, an IRP challenge was imposed upon B16-F10 tumor-bearing WT and hAAT^+/+^ mice. The resulting data demonstrate a unique response pattern to oxygen deprivation and subsequent rise of reactive oxygen species, as may be inferred from significantly greater upregulation of VEGF and IFNγ transcripts in tumors extracted from hAAT^+/+^ mice. These results signify the importance of context-dependent factors that may impact the function of hAAT.

Taken together, these results depict a role for hAAT as a tumor-inhibiting agent. This is the first study to examine the influence of hAAT over tumor development in immune-competent animals and provides important experimental evidence for the safety of hAAT therapy. The pro-inflammatory polarization of TAMs observed in hAAT-treated animals constitutes a novel concept, according to which hAAT acquires pro-inflammatory attributes in a context-dependent manner, most probably molecularly altered by the highly distinct conditions present in the tumor environment, including elevated NO production, oxygen deprivation, and the generation of free radicals. In order to fully understand the pro-inflammatory effects of hAAT in tumor settings versus the anti-inflammatory resolution-favorable attributes in other settings, continued studies in this intriguing field are required.

## Author Contributions

OG planned the study, performed the experiments, and wrote the manuscript. GF-L performed the experiments. ZK participated in planning of the study and performed the experiments. YL performed the experiments. PR participated in planning of the study. EL planned the study and wrote the manuscript.

## Conflict of Interest Statement

The authors declare that the research was conducted in the absence of any commercial or financial relationships that could be construed as a potential conflict of interest.

## Supplementary Material

Figure S1 **hAAT-treated animals retain similar short-term tumor cell clearance rates**. **(A,B)** C57BL/6 mice (*n* = 5 per group) were pretreated with hAAT (2 mg per animal) 24 h prior to inoculation with differentially stained RMA (stained with DiO) and either **(A)** RMA-S or **(B)** B16-F10 (stained with DiD) cells (i.v. 1:1 ratio, 1 × 10^6^ cells per animal). After 5 h, mice were sacrificed and cell ratios in the lungs were measured by flow cytometry. Mean ± SEM. Unpaired two-tailed Student’s *t*-test was employed to assess differences between groups.

Figure S2 **hAAT-treated mice display inhibited development of RMA, but not RMA-S tumors**. C57BL/6 mice (*n* = 5 per group) were pretreated with hAAT (2 mg per animal) and after 24 h prior to inoculation with differentially stained RMA (stained with DiO) and B16-F10 (stained with DiD) cells (i.v. 1:1 ratio, 1 × 10^6^ cells per animal). After 5 h, mice were sacrificed and cell ratios in the lungs were measured by flow cytometry. Mean ± SEM. Unpaired two-tailed Student’s *t*-test was employed to assess differences between groups. C57BL/6 mice (*n* = 6 per group) were pretreated with hAAT (2 mg per animal) 24 h prior to inoculation with RMA cells or RMA-S cells (1 × 10^5^ cells per animal, s.c.); hAAT was administered every third day afterward and tumor sizes were determined routinely. Mean ± SEM, **p* < 0.05. Unpaired two-tailed Student’s *t*-test was employed to assess differences between groups.

Figure S3 **Pro-inflammatory profile of TADCs in hAAT-treated tumor-bearing mice**. PBS- and hAAT-treated RMA tumor-bearing mice (*n* = 4 per group) were sacrificed 13 days after RMA cell inoculation (1 × 10^5^ cells per animal, s.c.). Tumors were dissociated, and cell populations were stained for indicated markers.

## Abbreviations

AAT, α1-antitrypsin; BMDM, bone marrow-derived macrophage; hAAT, human α1-antitrypsin; NK cell, natural killer cell; NO, nitric oxide; S-NO-hAAT, S-nitrosylated human AAT; TAM, tumor-associated macrophage.
