# Supplementary figures and images for: Context-Specific and Immune Cell-Dependent Antitumor Activities of α1-Antitrypsin
Source: Front Immunol. 2016 Dec 7;7:559. doi: 10.3389/fimmu.2016.00559 (PMC5141363; doi:10.3389/fimmu.2016.00559)

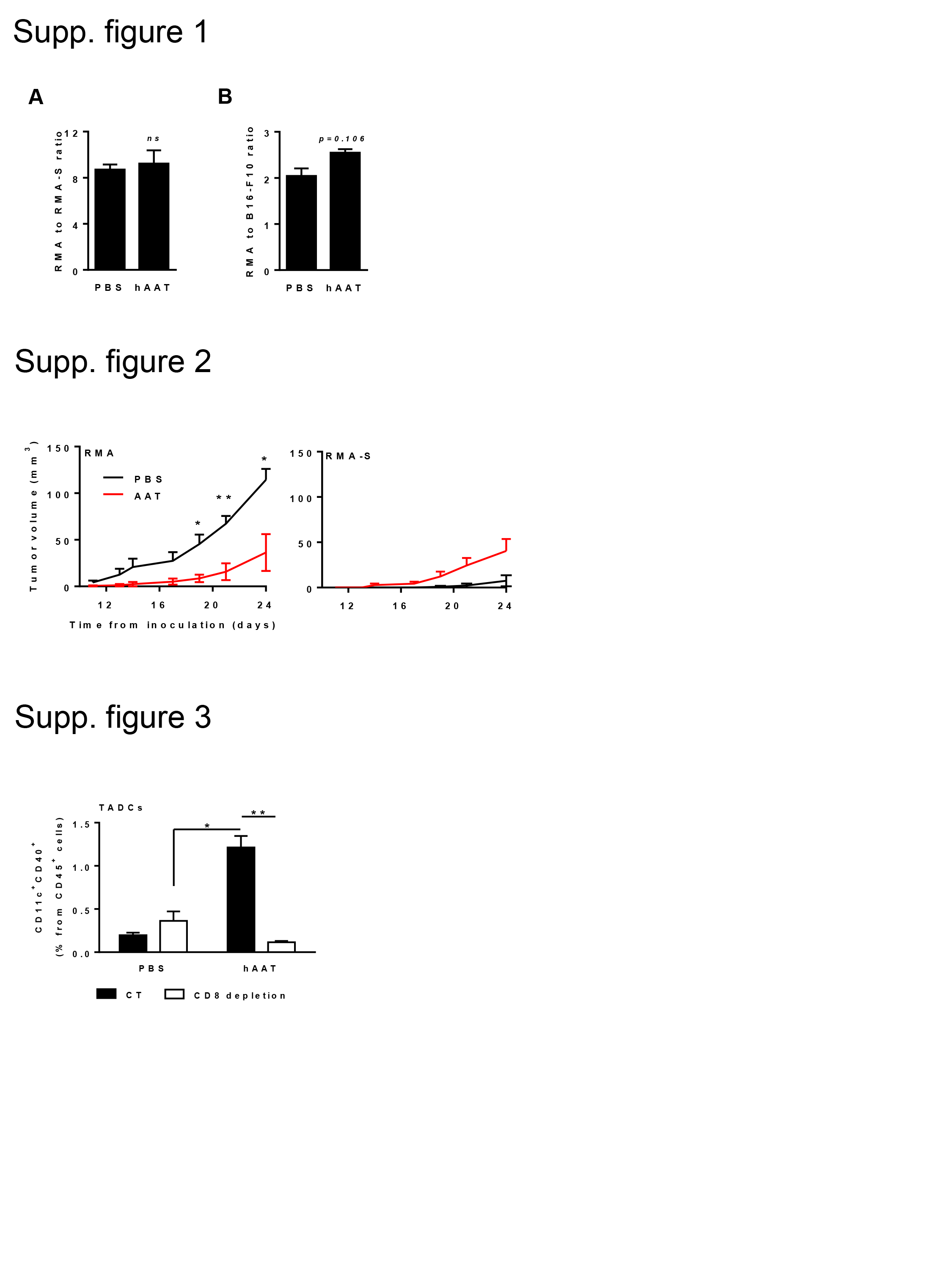

Supplement: Figure S1 — hAAT-treated animals retain similar short-term tumor cell clearance rates. (A,B) C57BL/6 mice (n = 5 per group) were pretreated with hAAT (2 mg per animal) 24 h prior to inoculation with differentially stained RMA (stained with DiO) and either (A) RMA-S or (B) B16-F10 (stained with DiD) cells (i.v. 1:1 ratio, 1 × 10^6^ cells per animal). After 5 h, mice were sacrificed and cell ratios in the lungs were measured by flow cytometry. Mean ± SEM. Unpaired two-tailed Student’s t-test was employed to assess differences between groups. [file Image_1.tif]
